# Attachment Security and Self-compassion Priming Increase the Likelihood that First-time Engagers in Mindfulness Meditation Will Continue with Mindfulness Training

**DOI:** 10.1007/s12671-016-0499-7

**Published:** 2016-02-29

**Authors:** Angela C. Rowe, Laura Shepstone, Katherine B. Carnelley, Kate Cavanagh, Abigail Millings

**Affiliations:** School of Experimental Psychology, University of Bristol, 12a Priory Road, Bristol, BS8 1TU UK; University of Southampton, Southampton, England; University of Sussex, Brighton, England; University of Sheffield, Sheffield, England

**Keywords:** Adult attachment, Security, Mindfulness, Self-compassion, Priming

## Abstract

Mindfulness practice has many mental and physical health benefits but can be perceived as ‘difficult’ by some individuals. This perception can discourage compliance with mindfulness meditation training programs. The present research examined whether the activation of thoughts and feelings related to attachment security and self-compassion (through semantic priming) prior to a mindfulness meditation session might influence willingness to engage in future mindfulness training. We expected both of these primes to positively influence participants’ willingness to continue with mindfulness training. We primed 117 meditation-naïve individuals (84 female; mean age of 22.3 years, SD = 4.83) with either a self-compassion, attachment security, or a neutral control prime prior to an introductory mindfulness exercise and measured their post-session willingness to engage in further training. Both experimental primes resulted in higher willingness to engage in further mindfulness training relative to the control condition. The self-compassion prime did so indirectly by increasing state mindfulness, while the attachment security prime had a direct effect. This study supports theoretical links between self-compassion and mindfulness and reveals a causal role for these factors in promoting willingness to engage in mindfulness training. Our findings have implications for improving compliance with mindfulness intervention programs.

## Introduction

Mindfulness is the ability to focus attention in the present whilst acknowledging that thoughts and emotions that spring to mind are fleeting and changeable (Kabat-Zinn 1990). It is a disposition (trait) but can be trained through meditation practice (Bishop et al. 2004; Chiesa 2013; Ives-Deliperi et al. 2011). Trait mindfulness correlates positively with the ability to endure uncomfortable emotions and feelings (Hayes and Feldman 2004), decreased impact of harmful emotional events (Kabat-Zinn 1990) and lower emotion reactivity (Baer et al. 2004).

Mindfulness meditation techniques vary but typically involve meditators adopting a sitting position and focusing their attention on an attentional anchor, such as the breath. When attention moves away from the anchor, as it inevitably does, the task is to acknowledge this without judgment and to re-direct attention back to the anchor. The aim is to develop a dispassionate and observant state of mind where thoughts and feelings that arise during meditation are seen as mental events without subjective value (Malinowski 2008).

Mindfulness training has many psychological and clinical benefits (Chiesa and Serretti 2009; Galante et al. 2014, Kabat-Zinn 1990; Kuyken et al. 2008), such as enhanced emotion regulation (Creswell et al. 2007; Ives-Deliperi et al. 2011). As such, a number of clinical and psychological interventions now include mindfulness training programs. Mindfulness-based interventions are clinically efficacious for a number of conditions. For example, mindfulness-based stress reduction (MBSR) has been successfully used to treat chronic pain and stress (Kabat-Zinn 1990), and mindfulness-based cognitive therapy (MBCT, mindfulness training combined with cognitive behavioural therapy) is successful in treating common mental health problems, such as depression and anxiety (Kuyken et al. 2008; Segal et al. 2004; Strauss et al. 2014). Mindfulness-based interventions have also been found to result in fewer negative automatic thoughts (Frewen et al. 2007), diminished anxiety (Shapiro et al. 1998), improved attention (Jha et al. 2007) and enhanced state self-esteem (Pepping et al. 2013).

Research aimed at specifying the psychological mechanisms by which mindfulness achieves its positive effects is growing, but this literature is rather limited. Neuro-imaging data show that mindfulness meditation lowers activation in brain areas such as the dorsolateral prefrontal cortex, the anterior cingulate cortex, the orbitofrontal cortex and the amygdala, all of which are associated with emotion regulation (Creswell et al. 2007; Ives-Deliperi et al. 2011), suggesting that the clinical benefits of mindfulness-based interventions are underpinned by the enhancement of positive emotion regulation strategies (Chiesa et al. 2014). Creswell and Lindsay (2014) propose a stress buffering account of how mindfulness works, such that mindfulness-based health effects are most evident in high-stress populations. Heightened self-compassion and decreased rumination and experiential avoidance have also been suggested as mechanisms by which mindfulness has its positive effects (Chiesa et al. 2014).

Although the clinical benefits of sustained mindfulness meditation are clear, not all those exposed to mindfulness training persist with it to the point where they derive benefit. Mindfulness meditation training is not easy. Despite the instruction to focus on an attentional anchor, such as breathing, thoughts and feelings commonly and repeatedly spring to mind. This could be perceived by some as evidence of ‘failure’ and may discourage persistence with training. In recognition of the potential difficulties with mindfulness meditation, it has been suggested that techniques, such as relaxation, may be used to counteract uncomfortable emotions as they arise in meditation (Dobkin et al. 2012). Individual differences in trait mindfulness might further exacerbate the problem, as for individuals who are low in trait mindfulness, achieving a mindful state is inherently more difficult than for others (Ives-Deliperi et al. 2011).

Self-compassion reflects a non-judgmental awareness of one’s inadequacies and failures (Neff 2003) and is strongly associated with psychological health (Neff and McGehee 2010). A number of mindfulness interventions include self-compassion training as an integral part of their programs. The cultivation of self-compassion in MBCT plays an important role in facilitating symptom change (Kuyken et al. 2008) and is increased by mindfulness, suggesting a clear link between self-compassion and mindfulness abilities (Birnie et al. 2010). Indeed, Neff (2003) proposed that mindfulness is essential for true self-compassion. Enhancing self-compassion in adults (through semantic priming) has positive effects, including increasing creativity and regulating eating behaviours (Adams and Leary 2007; Zabelina and Robinson 2010). Enhanced self-compassion may render individuals less critical of the self during meditation, which could be of particular utility when distractor thoughts and feelings appear. On this basis, the priming of self-compassionate thoughts and feelings prior to an initial mindfulness training session may make achieving a state of mindfulness easier and consequently increase willingness to persist with a mindfulness training program.

The activation of a sense of attachment security may also facilitate the mindfulness meditation process. Consistent experiences in important and long-term relationships result in mental representations of the self and others in relationships (Bowlby 1969). These attachment models are important predictors of affect regulation in response to threat (Mikulincer and Shaver 2001). Individual differences in these models are expressed in attachment orientations which vary on two orthogonal dimensions: *anxiety* about abandonment and *avoidance* of intimacy (Brennan et al. 1998). People high in attachment avoidance strive to maintain emotional distance from others, are compulsively self-reliant and have a positive view of the self while downplaying the value of close others. High avoidant individuals deactivate their attachment systems and suppress negative emotions, which is effortful. Those high in attachment anxiety have hyperactivated attachment systems and are vigilant for relationship threat cues, which is also effortful. They have unstable self-esteem and perceive others as unreliable (Brennan et al. 1998). Individuals who are secure in attachment have low levels of anxiety and intimacy avoidance and are happy to trust and depend on close others. When distressed or under threat, they can seek support from an external source or an internalised representation of a secure base, resulting in increased energy to engage in other behaviours and explore their environment because their attachment needs are met (Mikulincer and Shaver 2005). Most adults have cognitive access to both secure *and* insecure relationships (characterised by attachment anxiety and/or avoidance, Rowe and Carnelley 2003), in addition to a trait-like dispositional attachment style. This means that attachment security can be activated by priming to mirror (albeit temporarily) the effects of dispositional security. Indeed, a growing literature charts the positive psychological effects of security priming (Baldwin et al. 1996; Carnelley and Rowe 2007; Mikulincer and Shaver 2001).

Both secure individuals and those who are high trait mindfulness are efficient emotion regulators (Hayes and Feldman 2004; Mikulincer and Shaver 2001), and correlational studies show that dispositionally secure individuals report higher trait mindfulness than insecure individuals (Goodall et al. 2012). This suggests that attachment security and the ability to learn mindfulness may be directly related. We reason that the effects of an attachment security prime, while similar to those proposed above for a self-compassion prime, might additionally make it easier to put from mind-worrying interpersonal thoughts. Attachment security priming has been found to increase felt energy (a feeling of aliveness and vitality, Ryan and Frederick 1997), which in turn increased willingness to explore the environment (Luke et al. 2012). Felt energy may also, therefore, render people more open to explore novel experiences, such as meditation.

The psychological and health benefits of mindfulness training are increasingly clear. This makes it important to identify factors that might promote engagement with mindfulness training. We examined the effects of primed self-compassion and attachment security on willingness to engage in further mindfulness in mindfulness-naïve participants. We expected both primes to lead to higher willingness to engage in further training, relative to a neutral prime (direct effects). We expected that the effects of self-compassion would be mediated by state mindfulness and that the effects of the secure prime would be mediated by feelings of subjective energy (indirect effects).

## Method

### Participants

One hundred and seventeen individuals (84 female; mean age of 22.3 years, SD = 4.83) participated in the study. We based our sample size broadly on previous attachment orientation priming studies, where cell sizes are generally around 20 (Carnelley and Rowe 2007; Rowe and Carnelley 2003; Mikulincer and Shaver 2001). As the self-compassion prime is a relatively novel procedure, we nearly doubled the cell size (to 39 participants per cell). We stopped data collection once we had 39 participants per cell. Inclusion criteria are 18 to 35 years of age and no history of severe mental ill-health or of mindfulness training. Non-students (27 % of the sample) were entered into a prize draw for four separate prizes of £50. Students were awarded course credits.

### Procedure

Testing was done over a period of 4 weeks. Participants each attended one group session consisting of between 6 and 12 participants. On arrival at the lab, participants completed the Perceived Stress Scale (Cohen et al. 1983) and the Frieberg Mindfulness Inventory (Walach et al. 2006) in counterbalanced order. Participants were then randomly allocated to one of the three priming conditions (39 participants per condition). After the prime, participants assumed a comfortable position, while they listened to the recording of the mindfulness exercise. Participants next completed the manipulation check (felt security, compassion towards self, compassion towards others), mediator (state mindfulness, subjective energy) and criterion (willingness to engage in further mindfulness training) measures. A measure of attachment orientation was emailed to participants the following day. The delay was to ensure that dispositional attachment style would not cognitively interfere with the security prime (or vice versa). After the attachment measure was returned, participants were thanked and debriefed.

### Measures

#### Primes

The self-compassion prime (adapted from Rockliff et al. 2011) asked participants to ‘visualise and write about being completely compassionate and warm towards yourself’, while the secure attachment prime (adapted from Bartz and Lydon 2004) instructed participants to take some time to ‘visualise and write about a person with whom you have, or have had, a close secure relationship’. The neutral prime involved the visualisation of a recent and unaccompanied shopping trip undertaken by the participant (adapted from Carnelley and Rowe 2007). Participants wrote for 10 min about the prime theme on paper. They were told that they were free to take what they wrote with them. This instruction was designed to maximise the chances that participants would be uninhibited in what they wrote (Rowe and Carnelley 2003). The primes are available from the authors on request.

#### Mindfulness Meditation Exercise

The introductory mindfulness session was a pre-recorded audio file (10 min, 43 s), used in previous research (Erisman and Roemer 2010). It was an experiential exercise in which participants were given an explanation of mindfulness principles and how they might apply these to stay in the moment. Participants were then guided through mindful breathing for approximately 7 min.

#### Stress

The Perceived Stress Scale (PSS; Cohen et al. 1983) has 10 items (*α* = .77) measuring perceived stress over the previous month. Items include the following: ‘In the last month, how often have you felt that you were unable to control the important things in your life?’ and ‘In the last month, how often have you felt nervous and “stressed”?’. The response scale was 0 (*never*) to 4 (*very often*); scores were summed. As stress is commonly reduced in mindfulness-based clinical interventions (Astin 1997), we measured it as a potential covariate.

#### Trait Mindfulness

Trait mindfulness (a potential covariate) was measured using the Frieberg Mindfulness Inventory (FMI, Walach et al. 2006), a 14-item measure (*α* = .81) pertaining to experiences over ‘the last month’. Items include the following: ‘I pay attention to what’s behind my actions’ and ‘I feel connected to my experiences in the here and now’. The response scale was 0 (*rarely*) to 4 (*almost always*). Scores were summed.

#### Attachment Orientation

Attachment orientation (a potential covariate) was measured with an adapted (Rowe and Carnelley 2003) version of the Experiences in Close Relationships Scale (ECR; Brennan et al. 1998) designed to measure dispositional, as opposed to romantic, attachment orientation. The ECR measures the attachment dimensions of anxiety (18 items, *α* = .81) and avoidance (18 items, *α* = .87). The response scale for the ECR is 1 (*strongly disagree*) to 7 (*strongly agree*). Items include the following: ‘Just when people start to get close to me I feel myself pulling away’ and ‘I worry that people won’t care about me as much as I care about them’.

#### Felt Security

The Felt Security Scale (Luke et al. 2012) is a 16-item scale (*α* = .94) in which participants indicate how ‘comforted’ and ‘cared for’ (among other items) they feel at the given point in time on a scale from 1 (*not at all true*) to 7 (*very true*). Items were summed. Felt security was measured as a manipulation check for the primes (Carnelley and Rowe 2007; Rowe et al. 2012).

#### State Compassion Towards Self and Others

Compassion towards self and others was measured as a manipulation check for the primes using a visual analogue scale (VAS). Participants bisected 150-mm lines according to how much they agreed with each of the following questions: ‘I currently feel warm and compassionate towards others in my life’; ‘I currently feel warm and compassionate towards myself’ where 0 mm signified complete agreement and 150 mm indicated complete disagreement.

#### Willingness to Engage in Further Mindfulness Training

Willingness was measured using a VAS. Participants bisected a 150-mm line according to how much they agreed with each statement: ‘I would be willing to take up mindfulness in my own time’ where 0 mm identified complete agreement, whereas 150 mm indicated complete disagreement.

#### State Mindfulness

State mindfulness during the mindfulness session was measured (as a potential mediator) with the Toronto Mindfulness Scale (TMS; Bishop et al. 2006). The TMS is a 10-item (*α* = .84) measure. Items included the following: ‘I approached each experience by trying to accept it, no matter whether it was pleasant or unpleasant’ and ‘I was aware of my thoughts and feelings without over-identifying with them’. Items were rated on a 5-point scale from 0 (*not at all*) to 4 (*very much*).

#### Subjective Energy

We administered the Subjective Vitality Scale (Luke et al. 2012) as a potential mediator. The scale consists of 10-items (*α* = .93). Participants rated high energy descriptors such as ‘energetic, lively’ and ‘alive’ on a scale from 1(*not at all*) to 6 (*very much*) according to how they felt.

### Data Analyses

We estimated both the direct and indirect effects of each of our experimental primes using state mindfulness and energy as mediators in a multiple mediation model using PROCESS for SPSS (Hayes 2013). Our analysis estimates the direct effects of the self-compassion prime compared to the neutral prime, and the attachment security prime compared to the neutral prime, as well as the indirect effects of these primes through the mediators, state mindfulness and energy, on the criterion variable, willingness to engage in further mindfulness training.

## Results

Four ANOVAs were conducted on baseline variables and the ECR dimensions as preliminary analyses. These showed that trait mindfulness (*F*(2,114) = 2.25, *p* > .05) and stress (*F*(2,114) = .222, *p* > .05) did not vary by priming condition. Attachment anxiety (*F*(2,47) = 0.21, *p* > .05) and attachment avoidance (*F*(2,47) = 0.45, *p* > .05) did not vary by priming condition either. Neither of the baseline variables nor the attachment dimensions correlated with the main DV (see Table 1 for means and correlations for all variables). On the basis of these findings, neither the baseline variables trait mindfulness and stress nor the ERC dimensions were included as covariates in subsequent analyses. It should be noted that the ECR was sent to participants to complete after the experimental session, with a 41 % response rate. An ANOVA with prime and ECR completion (complete, did not complete) as the between participant factors and willingness to engage in further mindfulness meditation as the dependant variable showed no main effect of ECR completion, *F*(1,114) = .02, *p* = .88 and no prime by ECR completion interaction, *F*(2,114) = .77, *p* = .46. The low response rate meant that we did not have sufficient statistical power to examine the dimensions as moderators of the effects of prime.

**Table 1:** Descriptive statistics and correlations between all variables

Variable	Mean (SD, range)	1.	2.	3.	4.	5.	6.	7.	8.	9.	10
1. SM	29.76 (8.25) (47)	–	.02	.36**	.10	.32**	.12	−.14	−.63**	−.27**	−.50**
2. TM	34.05 (6.46) (34)		–	.32**	−.24	.28**	−.10	−.20	−.17	−.11	−.29**
3. FS	62.50 (14.85) (63)			–	−.26	−.60**	−.4	−.31*	−.39**	−.52**	−.64**
4. Stress	18.15 (6.78) (29)				–	−.26	.41**	.12	−.09	.01	.17
5. Energy	36.06 (11.39) (53)					–	−.21	.05	−.26**	−.43	−.42**
6. Anx.	3.40 (1.06) (4.4)						–	.33*	−.07	.05	.25
7. Avo.	3.23 (1.07) (4.9)							–	.15	.07	.34*
8. Will.	37.56 (28.11) (100)								–	.39*	.45**
9. Other comp.	24.15 (18.74) (93)										.79**
10. Self-comp.	34.65 (23.33) (100)										–

*N* = 117

*SM* state mindfulness, *TM* trait mindfulness, *FS* felt security, *Anx*. attachment anxiety, *Avo*. attachment avoidance, *Will.* willingness to engage in further mindfulness training, *Other Comp.* feelings of compassion for others, *Self Comp.* feelings of compassion for self

*p < .05; **p < .01

### Manipulation Checks

A MANOVA with prime as the between-participant factor and felt security, self-compassion and compassion towards others as the dependent variables, was significant, *F*(2,114) = 3.45, *p* < .01, *ηp*^2^ = .973, showing that felt security differed by condition, *F*(2,114) = 6.65, *p* < .01, *ηp*^2^=. 094, observed power = .870. Post hoc Tukey tests revealed that felt security scores were significantly higher in both the self-compassion (*M* = 66.33) and secure (*M* = 65.69) conditions than in the neutral (M = 55.84) condition (*p* < .01 and *p* < .05, respectively), although there was no significant difference between the two experimental conditions (*p* > .05).

Both compassion scores also differed by condition (lower endorsement indicates higher compassion). Self-compassion scores also differed by prime, *F*(2,114) = 4.547, *p* < .013, *ηp*^2^ = .074, observed power = .764. Post hoc Tukey tests revealed that self-compassion scores in the self-compassion (*M* = 29.07) and secure (*M* = 31.17) conditions were significantly lower (both *p < .*05) than in the neutral (*M* = 43.93) condition indicating higher self-compassion in the two experimental conditions. There was no significant difference between the two experimental conditions (*p* > .05). Compassion towards others was significantly lower (*F*(2,114) = 8.014, *p* < .001, *ηp*^2^ = .123, observed power = .952) in the experimental conditions relative to the neutral prime. Post hoc Tukey tests showed that ‘other compassion’ scores in both the secure attachment (*M* = 18.10) and self-compassion conditions (*M* = 21.28) (*p* < .01 and *p* < .05, respectively) were lower than the scores in the neutral condition (*M* = 33.31), indicating higher other compassion in the experimental conditions relative to neutral. The experimental prime conditions did not differ from each other (*p* > .05).

### Main Mediation Analyses

**Fig. 1:**
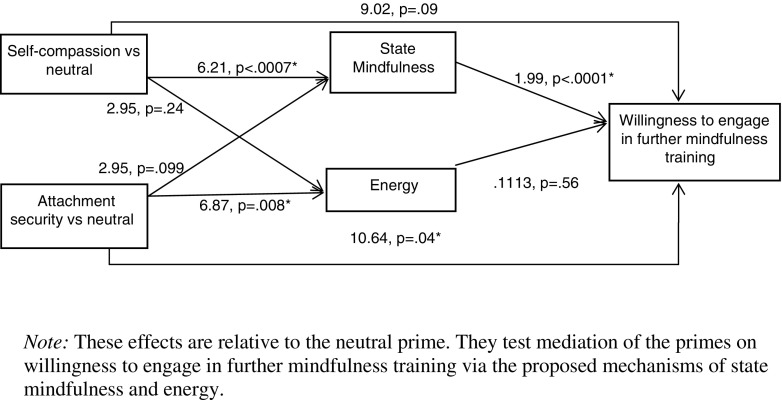
Full model depicting direct and indirect effects of self-compassion and attachment security primes (each compared to neutral) on willingness to engage in further mindfulness training

Our full model, depicting the coefficients for each path, is depicted in Fig. 1. The total and specific direct and indirect effects are described in the following text (Betas reported are unstandardised, and indirect effects are bootstrapped). As can be seen in Fig. 1, the self-compassion prime (compared to neutral) had a significant, positive effect on state mindfulness, *B* = 6.21, *SE* = 1.78, *t* = 3.50, *p* = .0007, 95 % CI = (+2.70, +9.72), and the attachment security prime (compared to neutral) had a significant positive effect on energy, *B* = 6.88, *SE* = 2.55, *t* = 2.70, *p* = .008, 95 % CI = (+1.83, +11.92).

The total effect of the self-compassion prime (compared to the neutral prime) on willingness to engage in further mindfulness training was significant, *B* = 21.68, *SE* = 6.10, *t* = 3.56, *p* = .0005, 95 % CI = (+9.61, +33.76). However, the direct effect of the self-compassion prime (compared to neutral) on willingness to engage in further training only reached a trend *B* = 9.02, *SE* = 5.21, *t* = 1.73, *p* = .09, 95 % CI = (−1.30, +19.35), whereas the total indirect effect was significant, *B* = 12.66, bootstrap *SE* = 4.01, bootstrap 95 % CI = (+5.35, +21.10). Specifically, the significance of the indirect effect was driven by the path via state mindfulness *B* = 12.33, bootstrap *SE* = 3.92, bootstrap 95 % CI = (+5.30, +20.54), rather than the path via energy *B* = .33, bootstrap *SE* = .76, and bootstrap 95 % CI = (−.52, +2.89). Thus, the self-compassion prime (compared to neutral) had a positive effect on willingness to engage in further mindfulness training, and this effect was entirely via increases in state mindfulness. Energy was not a significant mediator.

The total effect of the attachment security prime (compared to the neutral prime) on willingness to engage in further training was significant, *B* = 17.27, *SE* = 6.10, *t* = 2.83, *p* = .006, 95 % CI = (+5.20, +29.35). The direct effect of the attachment security prime (compared to neutral) was also significant, *B* = 10.65, *SE* = 5.12, *t* = 2.08, *p* = .04, 95 % CI = (+.49, +20.80). The total indirect effect of the attachment security prime (compared to neutral) was not significant *B* = 6.63, bootstrap *SE* = 3.96, bootstrap 95 % CI = (−.90, +14.57), and neither were the specific indirect paths via state mindfulness *B* = 5.86, bootstrap *SE* = 3.71, bootstrap 95 % CI = (−1.17, +13.40), or via energy *B* = .77, bootstrap *SE* = 1.37, bootstrap 95 % CI = (−1.28, +4.36). Therefore, the attachment security prime (compared to neutral) had a direct positive effect on willingness to engage in further mindfulness training. This effect was not mediated by either state mindfulness or energy.

To explore the potential effect of gender, we conducted an ANOVA with prime and gender as the between participant factors and willingness to engage in further mindfulness meditation as the dependant variable. Results show no main effect of gender, *F*(1,114) = 2.99, *p* = .90, and no prime by gender interaction, *F*(2,114) = .24, *p* = .79. However, it is worth noting that some cells were very sparsely populated (for example, there were only six males in the secure prime condition), so we treat these findings with caution.

Finally, to explore the differences in trait mindfulness, we conducted a regression analysis in which we used priming group, dummy coded as self-compassion (compared to neutral) and attachment security (compared to neutral) at step 1, trait mindfulness at step 2, and self-compassion (compared to neutral) X trait mindfulness and attachment security (compared to neutral) X trait mindfulness at step 3, to predict willingness to engage in further mindfulness mediation. Results showed that while priming, self-compassion (compared to neutral, *b* = 21.68, *p* = .001) and attachment security (compared to neutral, *b* = 17.27, *p* = .005) were significant predictors of willingness to engage in further mindfulness training, neither trait mindfulness (*b* = .48, *p* = .220) nor the interactions between trait mindfulness and (i) self-compassion (compared to neutral) (*b* = −1.93, *p* = .07) and (ii) attachment security (compared to neutral, *b* = −.67, *p* = .34) were significant.

## Discussion

We explored ways of maximising individuals’ willingness to continue with mindfulness training by priming self-compassion or attachment security. We reasoned that the self-compassion prime would render participants less damning of the self during meditation, in particular when thoughts and feelings sprung to mind as distracters. We reasoned also that the activation of security would render participants more positively disposed towards both the self and others, and worrying interpersonal thoughts would be easier to put from mind. We predicted that both of our experimental primes would make participants more willing to engage in further mindfulness training.

While both experimental primes were indeed found to increase willingness to engage in further mindfulness, they differed in the mechanisms that drove these effects. The attachment security prime had a significant direct effect and increased self-reported energy. The relationship between the prime and willingness to engage in further mindfulness training, however, was not mediated by energy. Although attachment security increases energy (Luke et al. 2012), neither energy nor state mindfulness appear to mediate between security priming and reports of willingness to further engage in mindfulness training in our data. This indicates that either attachment security directly results in greater willingness to engage in mindfulness training or that attachment security increases willingness to engage in further mindfulness training via an unmeasured mechanism, such as self-acceptance or calmness.

The self-compassion prime increased willingness to engage in further training indirectly, by increasing state mindfulness, as expected. Priming self-compassion is comparatively new in research; Leary et al. (2007) experimentally enhanced self-compassion by asking participants to think about a personal situation in a self-compassionate manner. The instruction to think self-compassionately across situations is a fairly common meditation activity (Hofmann et al. 2011) and psychotherapy technique (Gilbert 2009). We here show that self-compassion can be reliably primed with beneficial effects on willingness to continue with mindfulness training, suggesting that in combination with mindfulness training, self-compassion enhancement may render a challenging exercise (mindfulness meditation) more accessible.

Interestingly, trait mindfulness did not moderate the relationship between the primes and willingness to engage in further mindfulness training. This is intriguing as individuals with low trait mindfulness might have been expected to experience greater benefit from the primes. It should be noted though that our sample was relatively high in mean levels of trait mindfulness. Given this, a future research direction is to explore the effect of the security and self-compassion primes on willingness to engage in further mindfulness in a sample of individuals that are low in trait mindfulness.

A further avenue for future research might be to examine the effects of repeated priming of a sense of attachment security and self-compassion on actual meditation training program compliance over time. When primed repeatedly, security of attachment can have positive effects lasting a few days (Carnelley and Rowe 2007), even when delivered by time-efficient and practical methods such as texting (Otway et al. 2014). If a repeated priming methodology results in strong compliance to meditation training programs, it would have considerable implications for health outcomes.

### Limitations

The main study limitations were that we did not directly assess difficulties experienced during the mindfulness session and our dependent variable of ‘willingness to further engage in mindfulness training’ was a single-item measure. Future research might replicate the current procedure, but in addition, probe for any difficulties that may have been experienced during the mindfulness exercise, perhaps using funnelled debriefing with increasingly specific questions asked of participants about their experiences during the task. This would give an indication of how security and self-compassion priming might positively influence mindfulness engagement.

A further limitation could be the use of a single-item measure as a dependant variable in this study. Single-item measures are best used (a) for measuring unambiguous, one-dimensional constructs (Wanous et al. 1997) and (b) when a holistic impression is informative (Youngblut and Casper 1993). It is contended that this specification is highly appropriate for our dependant variable. Single-item measures have been used effectively to measure constructs as diverse as self-esteem (Robins et al. 2001), job satisfaction (Wanous et al. 1997) and readiness to change (Williams et al. 2007. They are as reliable as their multi-item counterparts (Robins et al. 2001) yet less onerous for participants to complete, therefore presenting clear advantages. Furthermore, single-item measures have predictive validity equivalent to multi-item measures (Bergkvist and Rossiter 2007; Nagy 2002). Despite the advantages of single-item measures, future studies might attempt to replicate our findings including a direct measure of difficulty experienced during meditation and a multi-item, or behavioural, measure of willingness to further engage in mindfulness training.
